# Inmunohistochemical detection of mastocytes in tissue from patients with actinic prurigo

**DOI:** 10.4317/jced.52823

**Published:** 2015-12-01

**Authors:** Eduwiges Martínez-Luna, Ronell Bologna-Molina, Adalberto Mosqueda-Taylor, Juan-Carlos Cuevas-González, Erika Rodríguez-Lobato, María-Abril Martínez-Velasco, María-Elisa Vega-Memíje

**Affiliations:** 1 Dr. Manuel Gea Gonzalez General Hospital, Dermatology, Mexico City; 2 Oral Pathology, Juarez University of Durango State; 3 Oral Pathology, University Autonoma Metropolitana, Mexico City

## Abstract

**Background:**

Actinic prurigo (AP) is a type of photodermatosis, the pathophysiology of which has not been determined. AP has been suggested to be a hypersensitivity reaction to the presence of eosinophils and the local production of IgE.

**Material and Methods:**

Descriptive study, using paraffin blocks of tissue that have been diagnosed with AP from the Dermopathology department, Hospital General Dr. Manuel Gea González. In 66 blocks from 63 patients, eosinophils were identified by hematoxylin and eosin staining, and mastocytes were labeled by immunohistochemistry. Three random microphotographs (40x) were used, and cell counts were calculated as the mean count in the 3 microphotographs.

**Results:**

Forty cases (63.5%) were female, and 23 (36.5%) were male. The mean age was 26.49 ±14.09 years; regarding the evolution time of the disease, the average was 11.93 years ±11.39. In 38 of 63 cases (60%), the lip, skin, and conjunctiva were affected clinically. In 22 of 63 cases (34%), AP cheilitis was the sole manifestation, and in 4 of 63 cases (6%), there were lesions in the skin and conjunctiva. The mean eosinophil count was 9 per case, the average number of mastocytes/field was 28.48 (range 0 to 66) Kruskal-Wallis *p*=0.001.

**Conclusions:**

There are elements in AP that mediate the reaction of hypersensitivity type IV b, necessitating the identification of triggering factors.

** Key words:**Actinic prurigo, eosinophil, hypersensitivity IV b, IgE, mastocytes.

## Introduction

Actinic prurigo (AP) is a chronic, inflammatory photodermatosis that affects the skin, lip, and conjunctival mucosa. AP primarily affects mixed-race and Amerindian populations who generally express the HLA-DR4 subtype DRB1*0407 allele (1-6) and live at altitudes over 1000 m above sea level (7).

AP usually develops in infancy and predominates in females up 4:1, (8-11) affecting photoexposed areas. Clinically, erythematous papules, excoriation, and hematic scabs that form plaques are observed; pruritus develops, causing areas of lichenification (9-11). Cheilitis is present in nearly 85% of AP cases, and in 27% of cases, it is the sole manifestation of the illness (12).

Between 45% and 62% of AP patients present with conjunctivitis and pseudopterygium formation (7,8,10). It evolves chronically, with partial remission (13). By histopathology, epithelial acanthosis, spongiosis and exulceration, and an abundance of eosinophils are seen in the dermis, and there is an infiltrate of nodular lymphoplasmocytes that can form lymphoid follicles, patognomonic image of AP´s chelitis (14,15). Dendritic follicular cells and plasma IgE-producing cells are also present, and IL-2 is produced by B and T lymphocytes (16). Elevated serum IgE has been reported in 10% to 50% of patients with AP and peripheral eosinophilia (17).

Ultraviolet radiation in AP, (18-22) effecting local lymphocyte proliferation through the secretion of soluble compounds by keratinocytes, with no change in serum leucocyte levels (23-25).

Mastocytes participate in inflammatory and allergic reactions. They are activated primarily through the high-affinity IgE receptor (FcεRI), which can bind the IgE-antigen complex to initiate a complex transduction of signals that culminate in the secretion of proinflammatory mediators and cytokines.

Other mechanisms include anaphylatoxins that generated by activation of the complement pathway, bacteria through Toll-like receptors, the release of TNF-α by mastocytes that amplify a stimulus, and T lymphocyte stimulation (26-29).

The presence of IgE-expressing eosinophils and mastocytes implicates a hypersensitivity reaction in the pathophysiology of AP (30). Generally, hypersensitivity reactions occur on exposure to an antigen through the activation of effector cells. There are 4 types of hypersensitivity reactions according the Gell and Coombs classification (31,32).

Hypersensitivity reaction type IV, or retarded reaction, is mediated specifically by T lymphocytes, which produce cytokines that activate various antibodies. The IVa subtype corresponds to a Th1 response, which stimulates macrophages by secreting IFNy in which complement-fixing antibodies and complement isotypes are co stimulants of proinflammatory and CD8 cells.

Conversely, the subtype IVb initiates a Th2 immune response in which lymphocytes T secrete IL-4, IL-5, and IL-13, which, through B cells that produce IgE and IgG4, stimulate eosinophils and mastocytes and inactivate macrophages. The high production of IL-5 causes eosinophilic inflammation, a characteristic of type IV hypersensitivity reactions (33-37).

The aim of this study was to examine the presence of mast cells and eosinophils in AP to increase our understanding of its pat-hophysiology.

## Material and Methods

This descriptive study we performed in the Dermopathology Department of Hospital General Dr. Manuel Gea González. The study samples were obtained from a pool of 40 tissue samples in skin with prurigo through a search of paraffin blocks using the clinical and histological criteria of AP.

From this database, we drew the demographics and characteristics of the patients whose tissues were included (sex, age, clinical localization, family records, and evolution time of the disease).

Using H&E slides for all cases, the diagnosis of AP was confirmed, and the presence of eosinophils and mastocytes was examined. The tissue block were, then slice and processed by immunohistochemistry. The tissues were deparaffinized, rehydrated, and subjected to antigenic recovery with 0.1% sodium citrate, pH 6.2, endogen peroxidase was inactivated (0.9% hydrogen peroxide), and the slides were incubated with 1% bovine serum albumin to block nonspecific sites. Washes were performed with PBS.

Tissues were incubated for 45 minutes with polyclonal anti-CD 117 (Dako1:50), after which anti-mouse/anti-rabbit secondary antibody was applied. Streptavidin-peroxidase complex was added to the slides for 30 minutes, and the reactions were visualized with diaminobenzidine (Dako) and counterstained with Hills´ hematoxylin.

The positive controls comprised 8 paraffin blocks of biopsies from various patients-2 healthy mucosal samples and 1 of each of the following: mucocele, venous lake, fibrous hyperplasia, traumatic cheilitis, sclerotic lichen, and granulomatous cheilitis.

Eosinophil counts and immunohistochemistry evaluation were performed semi-quantitative, based on positivity to anti-CD 117 (Dako1:50) in the membrane and cytoplasm of mastocytes. Three 40x microphotographs were selected randomly for H&E stai-ning for eosinophils and immunohistochemistry for mastocytes, all of them with and cells of 5x4, which include a surface of 0.3mm2. Counting of the cells present in each of the cells was performed and it was added respectively so the number of the total positive cells was able to be known in the whole image. Average counts were calculated over the 3 microphotographs.

## Results

Sixty-six blocks of tissue with a diagnosis of AP or AP cheilitis were selected. They had been diagnosed between 1995 and 2006 and contained enough biological material to be included in the study.

Forty cases (63.5%) were female, and 23 (36.5%) were male. The mean age was 26.49±14.09 (range 7-63 years).

The mean time in which the AP developed was 11.93 years ±11.39; time from diagnosis of AP ranged from 6 months to 45 years. Twenty-three patients (36.5%) had any immediate family member with a history of AP.

In 38 of 63 (60%) cases, the lip, skin, and conjuntiva were clinically affected; in 22 cases (34%), AP cheilitis was the sole manifestation (only used for the study tissue of AP located in skin). Four cases (6%) had lesions in the skin and conjunctiva but not in the lip.

**Figure 1:**
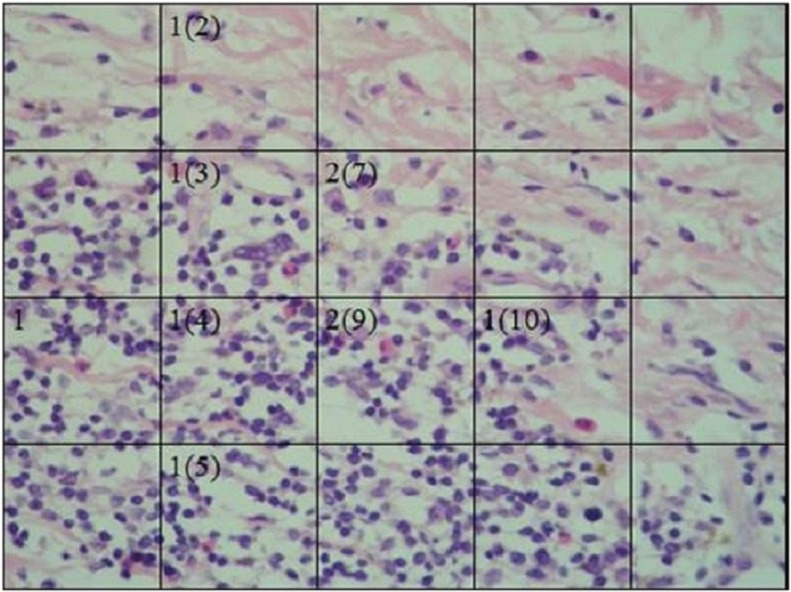
(HyE 40x) Presence of eosinophils in tissue with AP. Three micrographs were analyzed for each case, and the average of the 3 images was calculated.

In the 66 tissue samples that were diagnosed with AP and stained with H&E, the average number of eosinophils was 9 per case (Fig. 1). The average number of mastocytes in the mucosa of patients with AP was 28.48/field, ranging from 0 to 66 ( Table 1), versus 9.75/field in the control groups (range 5 to 16) and 7 mastocytes/field in healthy mucosa (Kruskal-Wallis *p*=0.001).

**Table 1:**
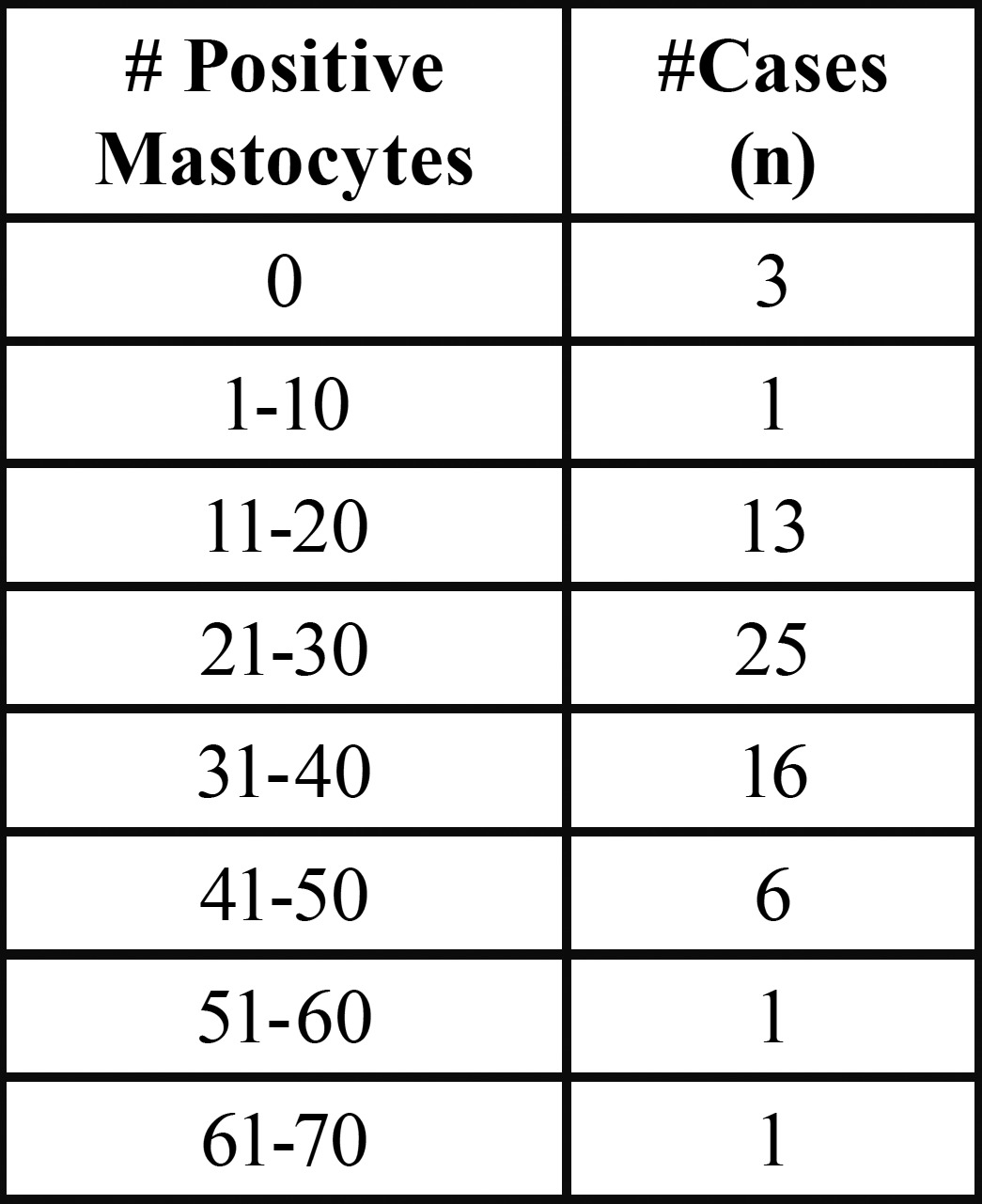
Number of positive mastocytes in AP samples.

**Figure 2:**
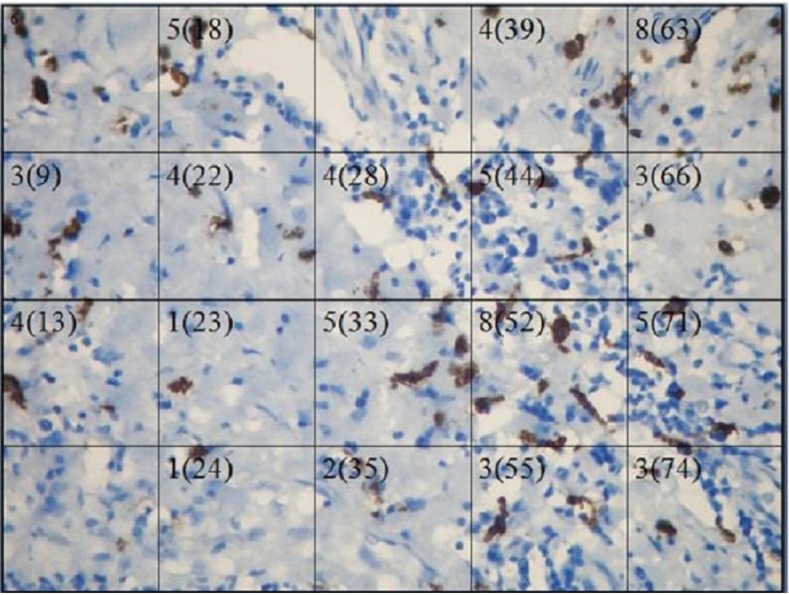
Membrane and cytoplasmic reaction of anti-CD117 in tissue with AP in skin.

Figure 2 shows representative immunohistochemistry staining of a randomly selected field. There were no significant differences in evolution time of disease or mean number of mastocytes per field. By calculation of Pearson´s correlation coefficient, we did not observe any linear relationship between mastocytes number and the evolution time of the disease ( Table 2) or between eosinophil number and the number of positive mastocytes.

**Table 2:**
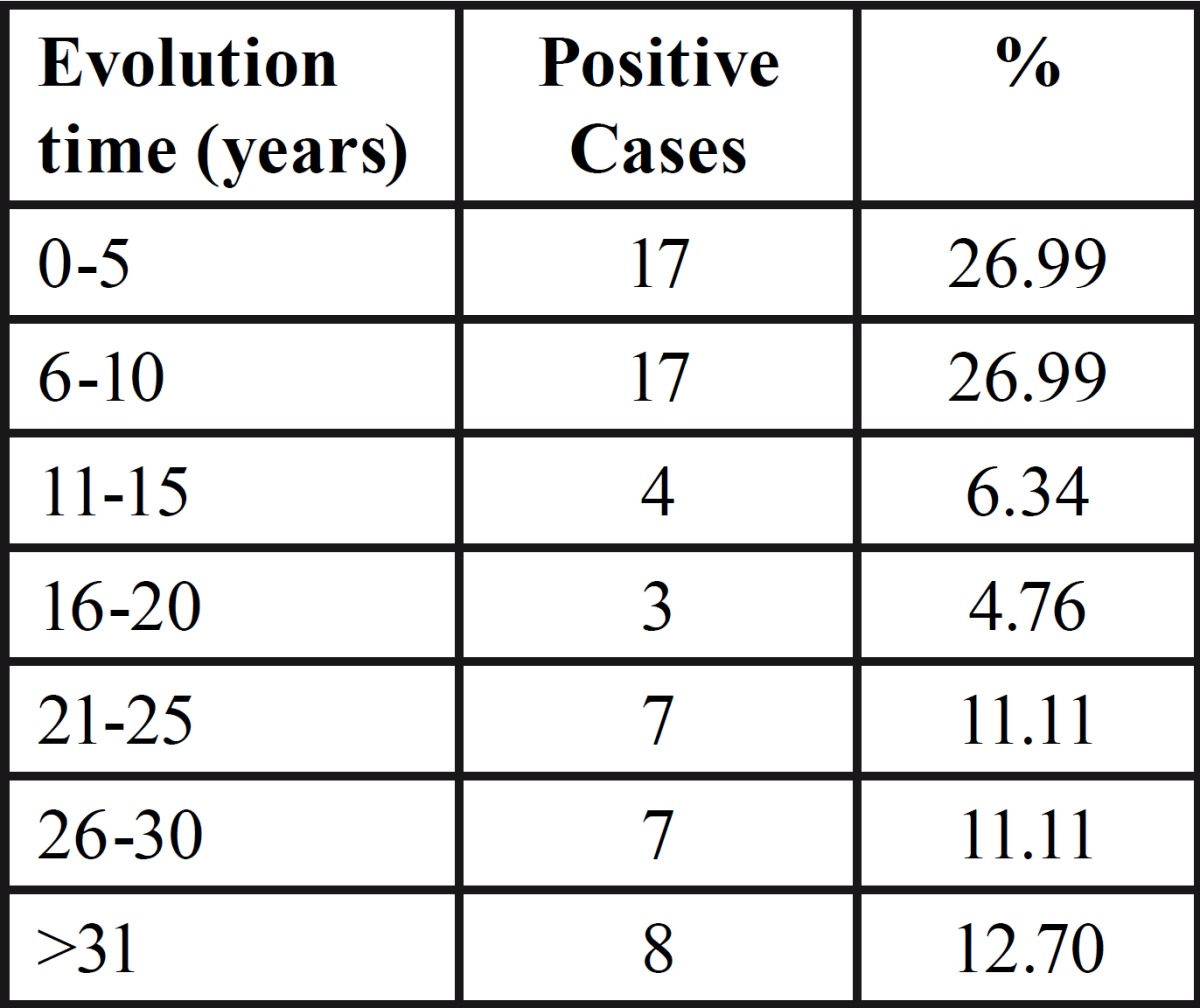
Evolution time of AP and number of positive mastocytes.

## Discussion

Actinic prurigo occurs predominantly in women. Although the first signs of which develop in childhood, patients are usually diagnosed late. At the time that the clinical history is taken, it is common to record data on any relatives with the disease (7,12-15,38). The characteristics of our study sample—a predominance of female patients, early age of onset (mean 14.35 years), and 36.3% of patients with a family history of PA-are consistent with these rates.

The number of mastocytes in normal skin increases from 44 to 75 mastocytes/mm2 and has an irregular distribution throughout the body, concentrating in the acral areas (39). In contrast, our AP patients had 189 mastocytes/mm2 at an enlargement of 40x and with a surface of 0.3 mm2.

The density of mastocytes per field was greater in mucosa of patients with AP versus healthy mucosa and other lesions, including those with an inflammatory origin, implicating these cells in the pathophysiology of AP. In contrast, there were no significant differences in the quantity of mastocytes or time of evolution.

Previous studies have reported the predominance of a Th2 response in AP, leading to the hypothesis that AP was a type I hypersensitivity reaction. In recent studies, however, when taking into account the presence of eosinophils, local production of IgE by plasma cells, increase in mastocytes number, detection of B and T cells, and participation of IL-2, an updated model suggests that the pathophysiology of AP is based on a type IV hypersensitivity reaction- specifically, subtype b-although subtype a can not be dismissed completely, based on these findings (16,24,36,39-41).

Future studies should attempt to identify an antigen that, with exposure to ultraviolet light, triggers an inflammatory reaction and leads to the clinical manifestations of AP.

## Conclusions

The higher density of mastocytes in samples from patients with actinic prurigo confirms the presence of all of the principal elements that mediate the reaction of late hypersensitivity type IV b, prompting us to hypothesize about the existence of a triggering allergen. More studies are required to resolve the pathophysiology of AP-specifically with regard to IL-2, IL-4, IL-5, and NK cells-and determine if and which apoptotic pathways are involved.

## References

[1] Granados J, Domínguez L (1993). Inmunogenética del prúrigo actínico en mexicanos. Dermatol Rev Mex.

[2] Dawe RS, Collins P, Ferguson J, O"Sullivan A (1997). Actinic prurigo and HLA-DR4. J Invest Dermatol.

[3] Hojyo T, Granados J, Vargas G, Yamamoto JK, Vega ME, Cortés R (1997). Further evidence of the role of HLA-DR4 in the genetic susceptibility to actinic prurigo. J Am Acad Dermatol.

[4] Wiseman MC, Orr PH, Macdonald SM, Schroeder ML, Toole JW (2001). Actinic prurigo: clinical features and HLA associations in a Canadian Inuit population. J Am Acad Dermatol.

[5] Menagé H, Vaughan RW, Baker CS, Page G, Proby CM, Breathnach SM (1996). HLA-DR4 may determine expression of actinic prurigo in British patients. J Invest Dermatol.

[6] Durán M (1993). Prúrigo actínico. Estudios de HLA. Antígenos de linfocitos humanos. Respuesta inmune celular. Dermatol Rev Mex.

[7] Hojyo T, Vega E, Granados J, Flores O, Cortés R, Teixeira F (1995). Actinic prurigo: an update. Int J Dermatol.

[8] Domínguez L (1993). Prurigo Actínico. Historia y situación actual. Dermatol Rev Mex.

[9] Novales J (1993). Prurigo actínico. Características clínicas. Dermatol Rev Mex.

[10] Magaña M (1997). Actinic or solar prurigo. J Am Acad Dermatol.

[11] Lane PR, Hogan DJ, Martel MJ, Reeder B, Irvine J (1992). Actinic prurigo: clinical features and prognosis. J Am Acad Dermatol.

[12] Vega-Memije ME, Mosqueda-Taylor A, Irigoyen-Camacho ME, Hojyo-Tomoka MT, Domínguez-Soto L (2002). Actinic prurigo cheilitis: clinicopathologic analysis and therapeutic results in 116 cases. Oral Surg Oral Med Oral Pathol Oral Radiol Endod.

[13] Hojyo MT (1993). Prurigo actinico. Diagnóstico diferencial. Dermatol Rev Mex.

[14] Vega ME, Ortega S, Hojyo MT, Reyes MM (1991). Queilitis. Correlación clínico-patológica. Dermatol Rev Mex.

[15] Herrera R, Magaña M (1995). Follicular cheilitis. A distinctive histopathologic finding in actinic prurigo. Am J Dermatopathol.

[16] Arrese JE, Domínguez L, Hojyo MT, Vega E, Cortés R, Guevara E (2001). Effectors of inflammation in actinic prurigo. J Am Acad Dermatol.

[17] Pizzi N (1993). Aspectos menos conocidos del Prurigo actínico. Dermatol Rev Mex.

[18]  Hojyo  MT,  Domínguez  L,  Magaña  M (1987). Phototesting in actinic prurigo. Volume of abstracts I.

[19] Hojyo MT (1993). Pruebas fotobiológicas en Prurigo actínico. Dermatol Rev Mex.

[20] Johnson JA, Fusaro RM (1998). Photosensitivity of the American Indian: Terminology and historical aspects. J Am Acad Dermatol.

[21] McGregor JM, Grabczynska S, Vaughan R, Hawk JL, Lewis CM (2000). Genetic modeling of abnormal photosensitivity in families with polymorphic light eruption and actinic prurigo. J Invest Dermatol.

[22] Epstein J (1993). La fotobiología del prurigo actínico. Dermatol Rev Mex.

[23] Ávalos E, Ramírez R, Vega ME (1993). ¿Es la luz ultravioleta el factor que desencadena la proliferación linfocitaria en la piel de los pacientes con Prurigo actínico?. Dermatol Rev Mex.

[24] Ávalos E, Ramírez R, Presno M (1993). Subpoblaciones de linfocitos T en pacientes con Prurigo actínico. Dermatol Rev Mex.

[25] Miyachi Y (1987). Reactive oxigen species in photodermatology. Photochem Photobiol.

[26] Miyazaki D, Nakamura T, Toda M, Cheung-Chau KW, Richardson RM, Ono SJ (2005). Macrophage inflammatory protein-1alpha as a costimulatory signal for mast cell-mediated immediate hypersensitivity reactions. J Clin Invest.

[27] Fischer M, Harvima IT, Carvahlo RF, Moller C, Naukkarinen A, Enblad G (2006). Mast cell CD30 ligand is upregulated in cutaneous inflammation and mediates degranulation-independent chemokine secretion. J Clin Invest.

[28] Atkins SR, Matteson EL, Myers J, Ryu JH, Bongartz T (2006). Morphological and quantitative assessment of mast cells in rheumatoid arthritis associated non-specific interstitial pneumonia and usual interstitial pneumonia. Ann Rheum Dis.

[29] Kanbe N, Tanaka A, Kanbe M, Itakura A, Kurosawa M, Matsuda H (1999). Human mast cells produce matrix metalloproteinase 9. Eur J Immunol.

[30] Chvatchko Y, Kosco MH, Herren S, Lefort J, Bonnefoy JY (1996). Germinal center formation and local immunoglobulin E (IgE) production in the lung after an airway antigenic challenge. J Exp Med.

[31] (1968). Classification of allergic reactions responsible for clinical hypersensitivity and disease. In: Clinical Aspects of Immnunology. 1a Ed.

[32] Warrington R, Silviu-Dan F (2011). Drug allergy. Allergy Asthma Clin Immunol.

[33] Pichler WJ (2003). Delayed drug hypersensitivity reactions. Ann Intern Med.

[34] Adam J, Pichler WJ, Yerly D (2011). Delayed drug hypersensitivity: models of T-cell stimulation. Br J Clin Pharmacol.

[35] Yu M, Tsai M, Tam S, Jones C, Zehnder J, Galli SJ (2006). Mast cells can promote the development of multiple features of chronic asthma in mice. J Clin Invest.

[36]  Abbas  AK,  Lichtman  AH (2004). Cellular and Molecular Immunology. 5a ed.

[37] Adam J, Pichler W, Yerly D (2011). Delayed drug hypersensitivity: models of T-cell stimulation. Br J Clin Pharmacol.

[38] Vega ME (1993). Características histopatológicas de Prurigo actínico. Dermatol Rev Mex.

[39] Janssens AS, Heide R, Hollander JC, Mulder PG, Tank B, Oranje AP (2005). Mast cell distribution in normal adult skin. J Clin Pathol.

[40] Moncada B, González R, Baranda ML, Loredo C, Urbina R (1984). Immunopathology of polymorphous light eruption. T lymphocytes in blood and skin. J Am Acad Dermatol.

[41] Umaña A, Gómez A, Durán MM, Porras L (2002). Lymphocyte subtypes and adhesion molecules in actinic prurigo: observations with cyclosporin A. Int J Dermatol.

